# An Unexpected Twist Behind Dysphagia in a Patient With a Goiter

**DOI:** 10.7759/cureus.113749

**Published:** 2026-07-31

**Authors:** Sanath N Rao, Mohan L N, Sharan Javali, Panchami P, Venkatesh Manoj

**Affiliations:** 1 Department of General Surgery, Vydehi Institute of Medical Sciences and Research Centre, Bangalore, IND

**Keywords:** aberrant right subclavian artery, ct aortogram, dysphagia, dysphagia lusoria, multinodular goiter, thyroidectomy, vascular anomaly

## Abstract

Dysphagia lusoria is a rare cause of dysphagia resulting from extrinsic esophageal compression by an aberrant right subclavian artery (ARSA). Its coexistence with thyroid pathology may obscure the diagnosis and delay appropriate management. We report a case of a 29-year-old female who presented with a multinodular goiter and dysphagia disproportionate to the size of the thyroid swelling. Clinical evaluation, thyroid ultrasonography, and fine-needle aspiration cytology confirmed a benign euthyroid multinodular goiter. Owing to persistent dysphagia, further evaluation was undertaken. A barium swallow demonstrated extrinsic compression of the esophagus, while computed tomography aortogram confirmed an ARSA coursing posterior to the esophagus, establishing the diagnosis of dysphagia lusoria. The patient underwent total thyroidectomy for the multinodular goiter; however, her dysphagia persisted postoperatively. She was subsequently referred to the cardiothoracic vascular surgery team for definitive surgical management of the ARSA. This case highlights the importance of considering vascular anomalies in patients with dysphagia that is disproportionate to the degree of thyroid enlargement. Early recognition through appropriate imaging and a multidisciplinary approach are essential for accurate diagnosis, avoidance of unnecessary interventions, and optimal patient outcomes.

## Introduction

Dysphagia lusoria is a term used to describe dysphagia as a result of compression of the esophagus due to an aberrant right subclavian artery (ARSA) [1]. The prevalence of an aberrant subclavian artery in the general population is estimated at 0.4% to 0.7% in the published literature [1]. We report a patient who had a multinodular goiter (MNG) with dysphagia caused by an ARSA. Difficulty in swallowing in thyroid disease may arise from compressive effects of goiter, malignant infiltration involving nearby nerves or structures, or treatment-related complications, while thyrotoxic myopathy represents an uncommon cause of dysphagia in hyperthyroid patients. However, the exact incidence of dysphagia in patients with a goiter is not known, although swallowing dysfunction in thyroid disease has been reported and may improve after thyroidectomy [2]. This report highlights the importance of good clinical evaluation and specific investigations to obtain a good outcome.

## Case presentation

A 29-year-old female presented with a thyroid swelling for four months, associated with difficulty in swallowing (more pronounced with solids than liquids) and a foreign body sensation in the neck. She denied any symptoms of dyspnea, voice changes, anorexia, or other gastrointestinal symptoms. There were no toxic symptoms. The patient had not received any treatment for the goiter earlier. There was no history of previous neck irradiation.

**Figure 1:**
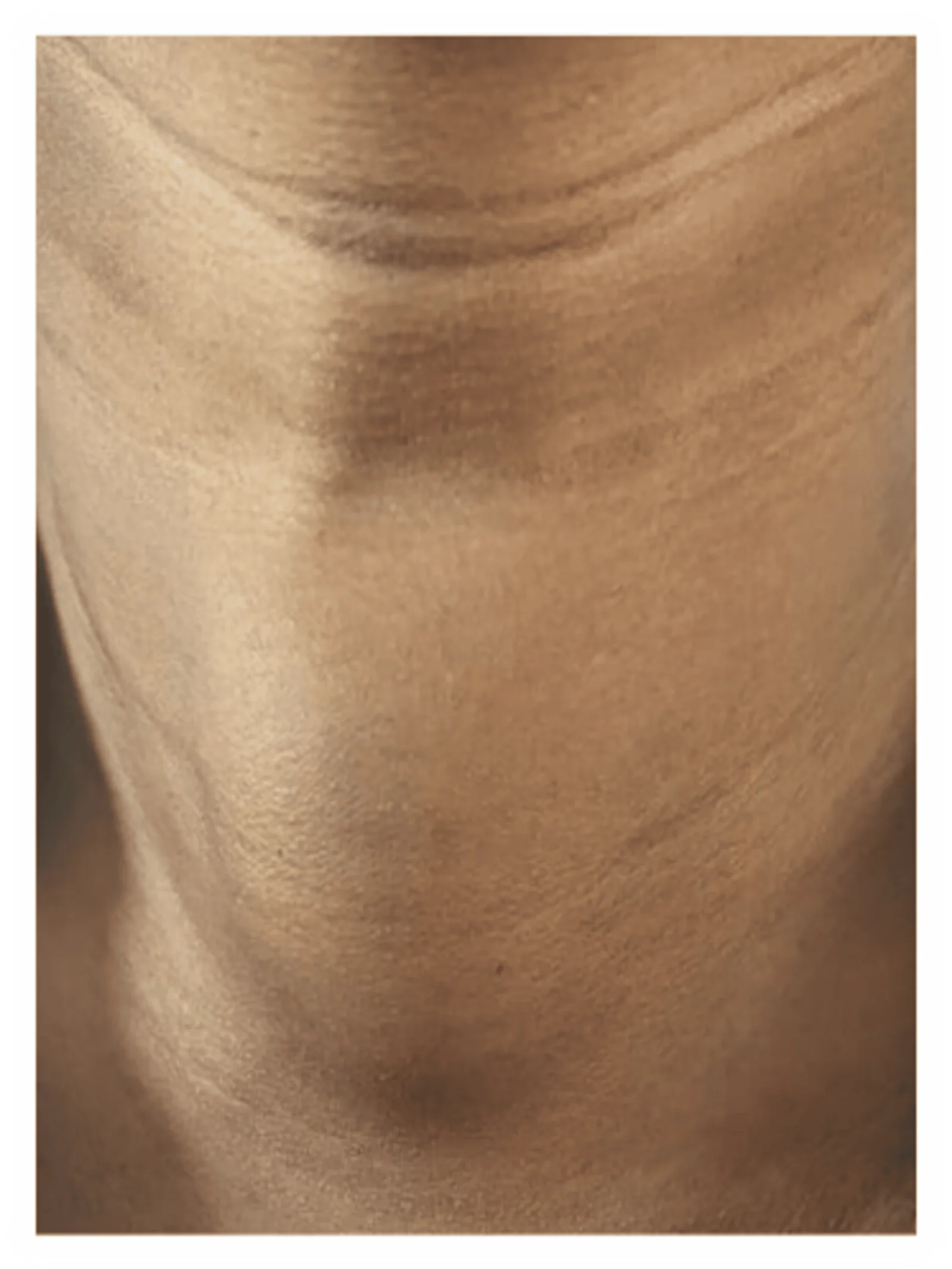
Clinical photograph of the patient.

On examination, her pulse rate was 88 bpm, and her blood pressure was 110/70 mmHg. Neck examination revealed an MNG involving both thyroid lobes, measuring approximately 4 x 3 cm, firm in consistency, and non-tender (Figure 1). No cervical lymphadenopathy was noted. The rest of the physical examination was within normal limits, with no features of toxicity. Her routine blood workup was within normal limits (Table 1).

**Table 1:** Thyroid test of the patient. The patient was in a euthyroid state.

Test	Patient’s value	Normal range
Thyroid-stimulating hormone	2.1	0.4-4.2
T3	1.53	0.7-2.04
T4	8.77	5.50-11
Free T3	3.01	2.3-4.2
Free T4	0.8	0.7-1.9

A diagnosis of euthyroid MNG was made, and investigations were performed.

**Figure 2:**
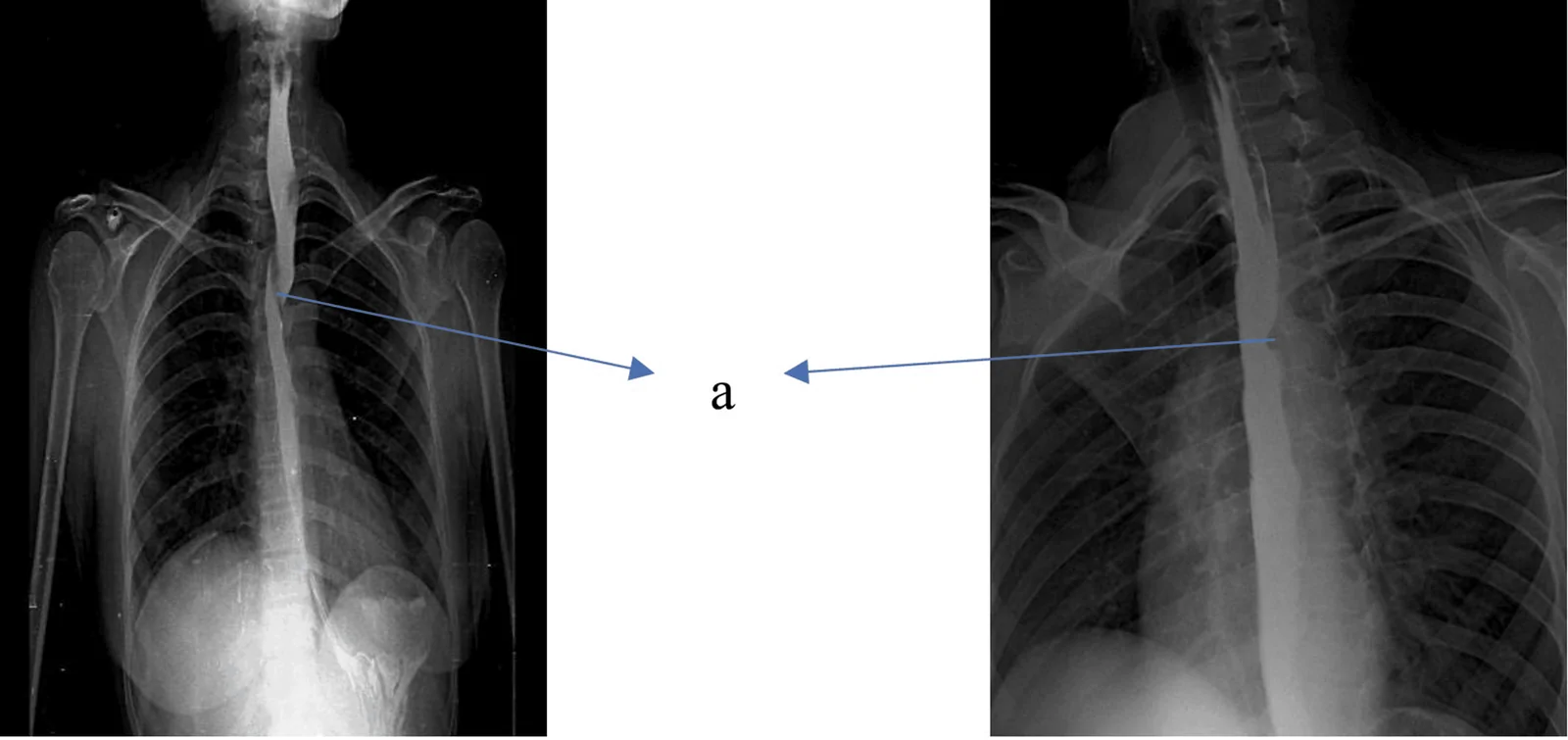
Barium swallow. (a) Extrinsic oblique compression of the esophagus at the level of T4. Aberrant right subclavian artery causing extrinsic compression of the esophagus.

Sonography of the neck revealed features suggestive of MNG, involving both lobes, the size of the largest nodule being 15 x 9.7 mm. Guided fine-needle aspiration cytology showed features suggestive of colloid goiter. X-ray of the neck was normal with no tracheal deviation. Since her dysphagia was out of proportion to the size of the thyroid, upper GI endoscopy and barium swallow were done (Figure 2). Upper GI endoscopy showed a normal mucosal study. Barium swallow showed an abnormal oblique extrinsic smooth pressure on the dorsal esophagus in the region of the T4 vertebra.

**Figure 3:**
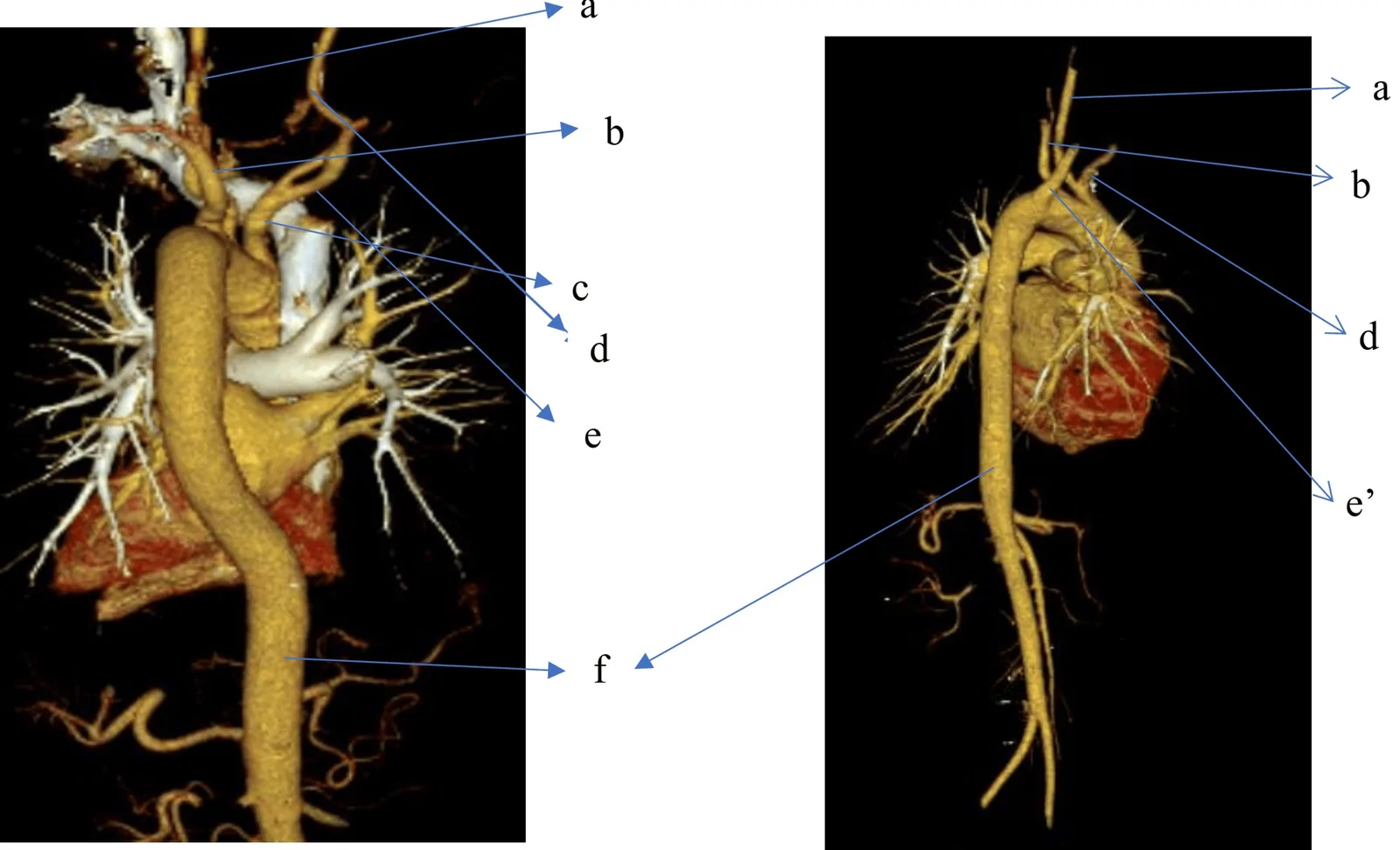
Normal aortogram (left) and aortogram showing aberrant right subclavian artery (right). a: left common carotid artery; b: left subclavian artery; c: brachiocephalic trunk; d: right common carotid artery; e: right subclavian artery; e’: aberrant right subclavian artery; f: descending aorta.

With a suspicion of ARSA, a CT aortogram was done, which confirmed the diagnosis. CT aortogram showed an ARSA arising from the arch of the aorta distal to the left subclavian artery at the level of T4 vertebra, seen running posterior to the esophagus, abutting and mildly displacing the esophagus to the left side. The right and left common carotid arteries originated from a common trunk arising from the arch of the aorta (bovine arch) (Figure 3).

The patient underwent total thyroidectomy under general anesthesia. The intraoperative finding was an MNG involving both lobes. Postoperative recovery was uneventful. She was discharged on replacement oral thyroxine.

Histopathological examination revealed features consistent with MNG complicated by chronic lymphocytic thyroiditis.

At a two-month follow-up, the patient continued to experience dysphagia and was referred to a cardiothoracic surgeon for evaluation and surgical management of the ARSA, which was the cause of her symptoms. The proposed procedure involved ligation of the ARSA, followed by re-anastomosis to either the left subclavian artery or the right common carotid artery.

## Discussion

Dysphagia lusoria is an unusual condition characterized by difficulty in swallowing secondary to compression of the esophagus by an ARSA. This condition is also known as Bayford syndrome. ARSA is a vascular anomaly involving the aortic arch [1]. It is hypothesized that a persistent seventh intersegmental artery with involution of the fourth vascular arch and right dorsal aorta leads to the formation of ARSA. The prevalence of ARSA is reported to be around 0.16%-2.0%, with dysphagia being the presenting symptom in approximately 20%-40% of cases [3].

Diagnostic tests such as a barium swallow have been used to screen for dysphagia lusoria, while CT or MRI helps define the vascular anatomy and plan surgical intervention [3]. Surgical management is indicated in patients with severe symptoms not manageable by conservative measures [4].

Our patient, a 29-year-old female with MNG, presented with dysphagia and a foreign body sensation in the neck. Although thyroid enlargement can cause compressive symptoms, dysphagia is less common unless the goiter is significantly enlarged [2]. In this case, the dysphagia was disproportionate to the thyroid size, prompting further evaluation. Additionally, the symptoms did not improve after thyroidectomy.

The key diagnostic finding on aortogram was the presence of an ARSA arising distal to the left subclavian artery and coursing posterior to the esophagus, causing esophageal compression. CT aortogram revealed a bovine arch variant, where the left common carotid artery shares a common origin with the brachiocephalic trunk, which has important clinical implications in vascular and surgical planning [5].

The patient underwent total thyroidectomy for MNG, followed by referral for cardiothoracic evaluation due to persistent dysphagia. Surgical management of ARSA typically involves ligation and revascularization, such as reanastomosis to the right common carotid or left subclavian artery, which effectively relieves esophageal compression [4].

A previously published case report by Pais Moreira et al. [6] described an incidental vascular anomaly identified during thyroid surgery. In that report, the ARSA was recognized because of its embryological association with a non-recurrent laryngeal nerve (NRLN), and the primary clinical significance was the prevention of recurrent laryngeal nerve injury during thyroidectomy. The patient did not present with dysphagia lusoria or symptomatic esophageal compression [6]. In contrast, our patient presented with a benign MNG and dysphagia that was disproportionate to the size of the thyroid swelling. Although thyroidectomy was performed for the MNG, persistent postoperative dysphagia prompted further evaluation, leading to the diagnosis of dysphagia lusoria caused by an ARSA. Unlike the previous report, where the vascular anomaly had primarily surgical implications, our case highlights the clinical significance of ARSA as an independent cause of dysphagia and underscores the importance of considering vascular anomalies in patients with persistent or unexplained dysphagia despite coexisting thyroid pathology.

Aberrant right subclavian artery and dysphagia lusoria

ARSA is a rare congenital vascular anomaly in which the right subclavian artery arises distal to the left subclavian artery from the aortic arch and typically courses posterior to the esophagus, leading to extrinsic compression [3]. This anatomical variation can result in dysphagia (dysphagia lusoria), particularly when the artery causes significant esophageal compression [3].

Dysphagia lusoria is an uncommon but important differential diagnosis in patients presenting with swallowing difficulty, especially when symptoms are disproportionate to structural abnormalities such as thyroid enlargement. In the present case, although the patient had an MNG, the severity of dysphagia prompted further evaluation, leading to the identification of ARSA on CT aortogram.

Management and surgical treatment

Management of dysphagia lusoria depends on the severity of symptoms. Mild cases may be managed conservatively with dietary modification and swallowing techniques. However, patients with persistent or severe symptoms require surgical intervention [4].

Surgical treatment of ARSA aims to relieve esophageal compression and restore normal vascular anatomy. The standard approach involves ligation of the aberrant artery followed by revascularization, typically by reanastomosis to the right common carotid artery or the left subclavian artery [4]. This procedure effectively alleviates symptoms by eliminating the compressive effect on the esophagus.

In this case, the patient initially underwent total thyroidectomy for the MNG. However, persistence of dysphagia postoperatively necessitated referral for cardiothoracic surgical evaluation and definitive management of ARSA.

## Conclusions

This case illustrates the importance of considering other causes of dysphagia, particularly vascular anomalies such as an ARSA, as a potential cause of dysphagia in patients with thyroid disease. While the thyroid enlargement in this patient was significant, it was the ARSA that was responsible for the disproportionate symptoms. This case highlights the need for a comprehensive diagnostic workup, including vascular imaging, when unusual or unexplained symptoms persist in patients with thyroid disorders. A multidisciplinary approach, including input from both endocrinologists and cardiothoracic surgeons, is essential in effectively managing such complex cases.

Here, we have compared our case, an MNG with symptomatic dysphagia lusoria, with a case of MNG with asymptomatic dysphagia lusoria, as the two conditions require different management approaches.
